# Lysozyme is Sterically Trapped Within the Silica Cage in Bioinspired Silica–Lysozyme Composites: A Multi-Technique Understanding of Elusive Protein–Material Interactions

**DOI:** 10.1021/acs.langmuir.2c00836

**Published:** 2022-06-23

**Authors:** Francesco Bruno, Lucia Gigli, Giovanni Ferraro, Andrea Cavallo, Vladimir K. Michaelis, Gil Goobes, Emiliano Fratini, Enrico Ravera

**Affiliations:** † Magnetic Resonance Center (CERM), University of Florence, via L. Sacconi 6, Sesto Fiorentino 50019, Italy; ‡ Department of Chemistry “Ugo Schiff”, University of Florence, via della Lastruccia 3, Sesto Fiorentino 50019, Italy; § Consorzio per lo Sviluppo dei Sistemi a Grande Interfase (CSGI), via della Lastruccia, 3, Sesto Fiorentino 50019, Italy; ∥ CERTEMA S.c.a.r.l., S.P. Del Cipressino Km 10, Cinigiano 58044, Italy; ⊥ Department of Chemistry, University of Alberta, Edmonton Alberta T6G 2G2, Canada; # Department of Chemistry, Bar-Ilan University, Ramat Gan 5290002, Israel; ¶ Consorzio Interuniversitario Risonanze Magnetiche di Metalloproteine (CIRMMP), via L. Sacconi 6, Sesto Fiorentino 50019, Italy

## Abstract

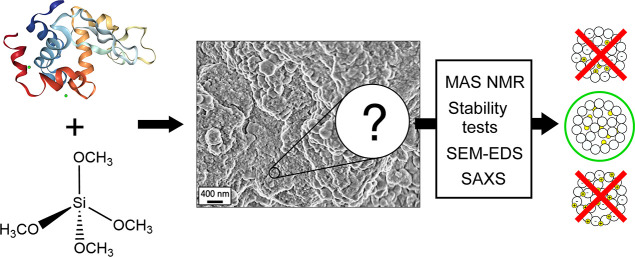

Lysozyme is widely known to promote the formation of condensed silica networks from solutions containing silicic acid, in a reproducible and cost-effective way. However, little is known about the fate of the protein after the formation of the silica particles. Also, the relative arrangement of the different components in the resulting material is a matter of debate. In this study, we investigate the nature of the protein–silica interactions by means of solid-state nuclear magnetic resonance spectroscopy, small-angle X-ray scattering, and electron microscopy. We find that lysozyme and silica are in intimate contact and strongly interacting, but their interaction is neither covalent nor electrostatic: lysozyme is mostly trapped inside the silica by steric effects.

## Introduction

Spherical silica is one of the most relevant contemporary industrial inorganic compounds, with uses that range across all areas of modern chemistry.^1−3^ With its large use, the sustainability and the environmental footprint of its production are to be considered. Bioinspired silica preparation (i.e., the formation of silica templated by biological macromolecules) has attracted an increasing interest being remarkably cost-effective,^4^ while tunability of the structure and reactivity of the silica-based composites is accessible through selection of biomolecules, offering a wide range of opportunities in several fields.^5,6^ Among the different bioinspired silica preparation strategies,^7,8^ the bioinspired synthesis proposed by Luckarift et al.^9^ is quite convenient. This synthesis uses lysozyme, a small prolate protein with a high positive surface charge, as a catalyst for the polycondensation of silicic acid. The latter is obtained from hydrolysis of silicic alkoxides such as tetramethylorthosilicate (TMOS). Therefore, the preparation is based on inexpensive^10^ and readily available reagents, is fast, can be performed under ambient conditions (i.e., room temperature and atmosphere), and yields a condensed silica network without thermal processing. Other inorganic oxides, such as titania, can be produced through a similar chemistry.^9^

Despite the significant interest in this approach, the templating effect of the protein on the silica aggregation mechanism is not completely understood. Lysozyme enhances the nanoparticle formation and affects the composite structure and composition,^11^ but a complete structural characterization of these silica–protein composites is still lacking. The reactivity of lysozyme, which is common to other highly positively charged proteins and polypeptides, is reasonably attributable to electrostatic interactions, which involve the positively charged side chains of lysozyme and the negatively charged monosilicic acid, oligomeric silica, and colloidal silica. Indeed, a recent crystal structure localized a hydrolyzed species of the titanium precursor at a positive patch on the protein surface.^12^ However, no such interaction could be observed for silicic acid.^12^ A recent study suggests that liquid–liquid phase separation (LLPS) is responsible for driving the polymerization; however, the LLPS is related to overall charge,^13^ and there are reports that lysozyme tends not to undergo LLPS at pH values around 7.^14^ It has been previously demonstrated that lysozyme catalyzes the silica colloid formation from silicon alkoxide solutions and dilute water glass solutions around neutral pH.^7,15^ In the Luckarift et al. work, it was demonstrated that the protein maintains its catalytic activity and is not removed by repeated washes.^9^ The latter indicates that the protein interacts strongly with silica. A recent study on both adsorption and co-precipitation experiments suggests that lysozyme neither alters the silica structure nor is trapped inside the silica.^16^ The same study states that partial unfolding of the enzyme can occur, and time-resolved SAXS data suggest that the structure of lysozyme is strongly distorted during the initial events of the composite formation and then regains its “native-like” shape at a later stage during the silica aggregation process.^17^ However, no evidence of structural distortion has been observed by solution nuclear magnetic resonance (NMR) spectroscopy.^12^

Lysozyme is often used as a model for interface interactions because it is easily obtainable, has intriguing biophysical properties (often proteins are negative, rather than positive^18^), and has a useful antimicrobial activity.^19−24^ Therefore, it is expected that an improved understanding of lysozyme–material interactions could provide hints for applications beyond the study of bioinspired mineralization.^25−27^

In the present work, we investigate this peculiar interaction occurring at the protein–material interface in the lysozyme–silica hybrid composite using solid-state NMR under magic-angle spinning (MAS), scanning electron microscopy (SEM), and small-angle X-ray scattering (SAXS). As these hybrid interfaces are non-crystalline in nature, MAS NMR spectroscopy is the ideal tool to unravel short- (1.5–5.0 Å) and medium-range (5.0–10.0 Å) atomic-level distances that define their structure.^28^

Solid-state NMR is indeed a critical methodology in the characterization of biomacromolecular interfaces, as demonstrated, for instance, in the characterization of bones,^29−33^ corals,^34^ and other biomaterials and bioinspired materials in general^35−37^ and silicic materials in particular.^38−40^

## Results and Discussion

The protein immobilization was carried out according to the protocol previously reported by Luckarift et al.^9^ A sample containing particles with spherical shape was obtained (SEM micrographs in Figure S1a). Elemental analysis using energy-dispersive X-ray spectrometry (EDS, Figure S1b) shows that nitrogen (arising from the protein) and silicon (arising from the matrix) are both present in the sample.

According to the previous literature,^11,16,17^ the possible relative arrangements of the protein and silica are:

(1) charge inside: the protein is trapped inside the silica particles by electrostatic interactions,
(2) steric inside: the protein is trapped inside the silica particles during their formation and cannot escape, while marginal interactions exist, and
(3) charge outside: the protein is interacting with the exterior of the silica particles via (mainly) electrostatic interactions.

**Scheme 1:**
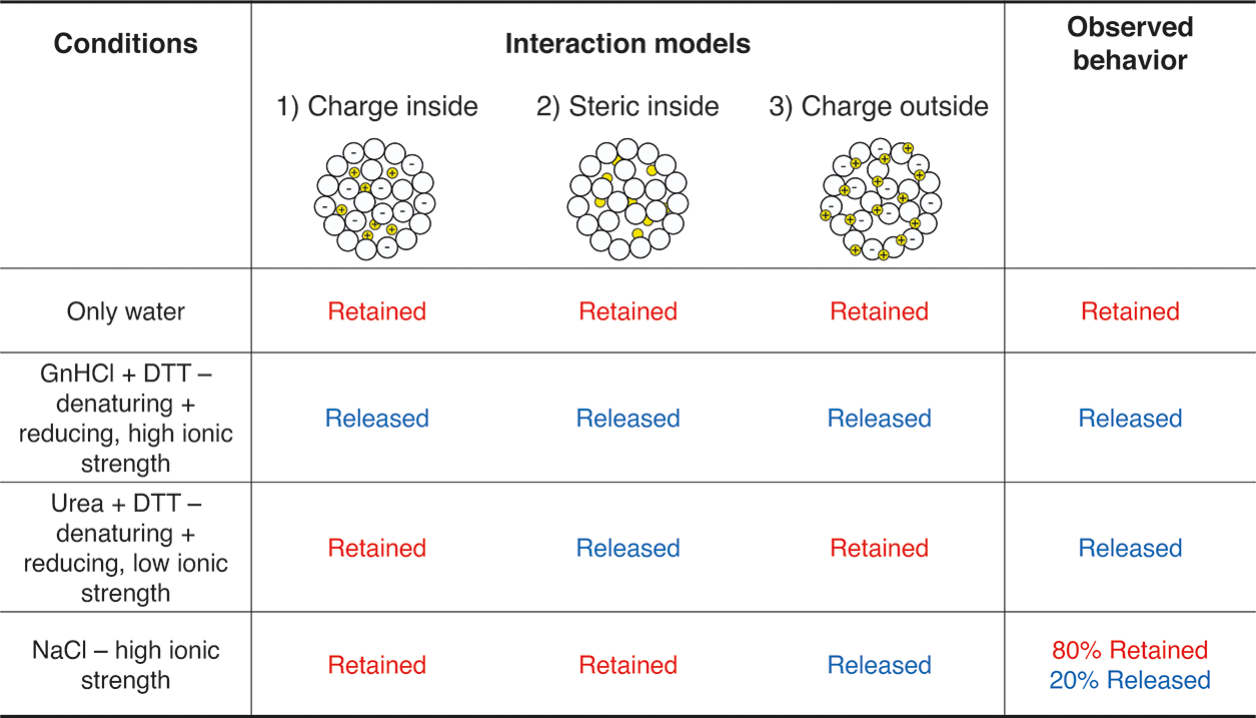
Behavior of the Composite With Respect to Ionic Strength and Denaturation

These three arrangements would lead to a distinct outcome for different treatments of the sample (Scheme 1). We find that, upon denaturation with either guanidinium chloride (GnHCl) or urea and reduction with dithiothreitol [(2*S*,3*S*)-1,4-bis(sulfanyl)butane-2,3-diol, DTT], the protein is quantitatively released, regardless of the ionic strength, leaving the silica structure with a porosity on the nanometer scale (SEM micrographs in Figure S2a). The removal of the protein from the inorganic network is confirmed by EDS results (Figure S2b), showing the disappearance of the nitrogen signal with respect to the as-prepared sample. Washing with 1 mol dm^–3^ sodium chloride causes the release of about 1/5 of the protein, as observed from the UV absorbance of the supernatant and confirmed by the decrease in the amount of nitrogen with respect to silicon at the end of the washing step (SEM micrographs and EDS spectrum in Figure S3a,b, respectively). In this last case, the morphology of the sample remains almost unaltered if compared to the freeze-dried starting composite. These experimental observations appear to favor situation #2, at least for 80% of the protein, whereas the remaining 20% may fall in situation #3.

**Figure 1:**
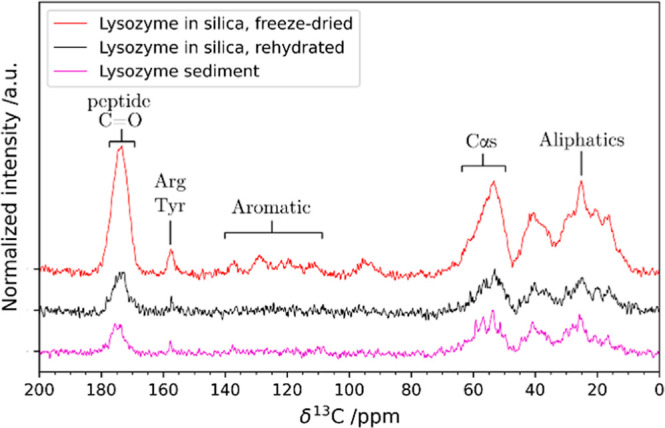
{^1^H}–^13^C CP MAS NMR spectra of the composite (red: freeze-dried and black: rehydrated) and of lysozyme sediment (magenta). The spectral regions where protein signals are usually observed are annotated on the spectra.

We have therefore proceeded with the solid-state NMR characterization (see details in the Supporting Information and Table S1). Observation of the protein resonances is possible through ^13^C MAS NMR spectra (Figure 1). These were first acquired for the freeze-dried sample. As typical for dry proteins, the resolution of the signals is low due to structural heterogeneity. However, upon rehydration, the spectral resolution increases because of the water that promotes local dynamics. This observation is rather common in solid-state NMR on passing from lyophilized to rehydrated biomolecules.^41−54^ The comparison with the spectrum of the sedimented lysozyme shows that the broader envelope of the composite spectrum encompasses the frequency range that is observed for the sedimented protein. Since the spread of the ^13^C NMR signals indicates the folding of the protein, we can infer that the fold of the protein is preserved.^55,56^ Even with a resulting broad spectrum, the distinctive features of the folded protein are observed, that is, features from the methyl-bearing side chains, which have shifts lower than 20 ppm when the hydrophobic core of the protein is intact, and in the overall distribution of the aliphatic region (65 to 5 ppm). This observation confirms previous reports by Mirau^57^ and by Ravera et al.^56^ and is in contrast with the idea that lysozyme loses its tertiary structure when the silica matrix is formed.^16^

It is worth noting that the peak around 157 ppm can be attributed to either arginine or tyrosine C_ζ_. Since the lysozyme sequence features 11 arginine and 3 tyrosine residues, most of the intensity of this peak can be attributed to arginine residues.^58^ The intensity increase of the peak in the freeze-dried composite with respect to the protein sediment can be attributed to a lower mobility of the side chain in the composite with respect to the free protein. We also note that this peak is markedly narrowed upon rehydration and, considering the high arginine content, this behavior is similar to what was observed for arginine ^15^N lines in ubiquitin interacting with MCM41.^54,59^

**Figure 2:**
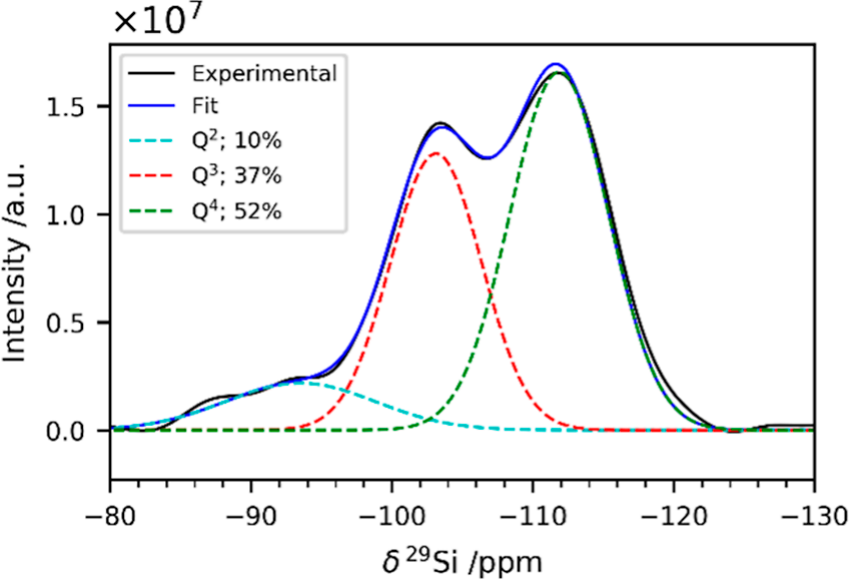
^29^Si direct-excitation MAS NMR spectra of the freeze-dried composite. The signals have been deconvoluted using three Gaussian peaks corresponding to *Q*^2^ (cyan), *Q*^3^ (red), and *Q*^4^ (green) sites.

Solid-state NMR also offers a unique view for the characterization of the silica matrix. The ^29^Si MAS NMR spectra show signals originating from siloxanes (*Q*^4^, around −110 ppm), single silanols (*Q*^3^, around −100 ppm), and geminal silanols (*Q*^2^, around −90 ppm), the latter being barely visible (Figure 2, refer to Scheme S1 for a summary of the observable species). The broad line widths are expected for amorphous silica as they are mainly affected by the dispersion of isotropic chemical shifts arising from a distribution of bond angles and distances.^60^ The results of the deconvolution are given in Table S2. The ratio *Q*^4^/*Q*^3^, which is indicative of the degree of condensation of the silica matrix, is 1.39, lying around the values observed for PL-12 templated bioinspired silica (1.54)^61^ and SBA-15 silica (1.60)^62^ and similar to the results obtained by Martelli et al., where similar preparation conditions were used.^63^ Notably, silica gel obtained under the same conditions but without the protein has a ratio of 0.68, and therefore, it is much less condensed (Figure S4, Table S3).

**Figure 3:**
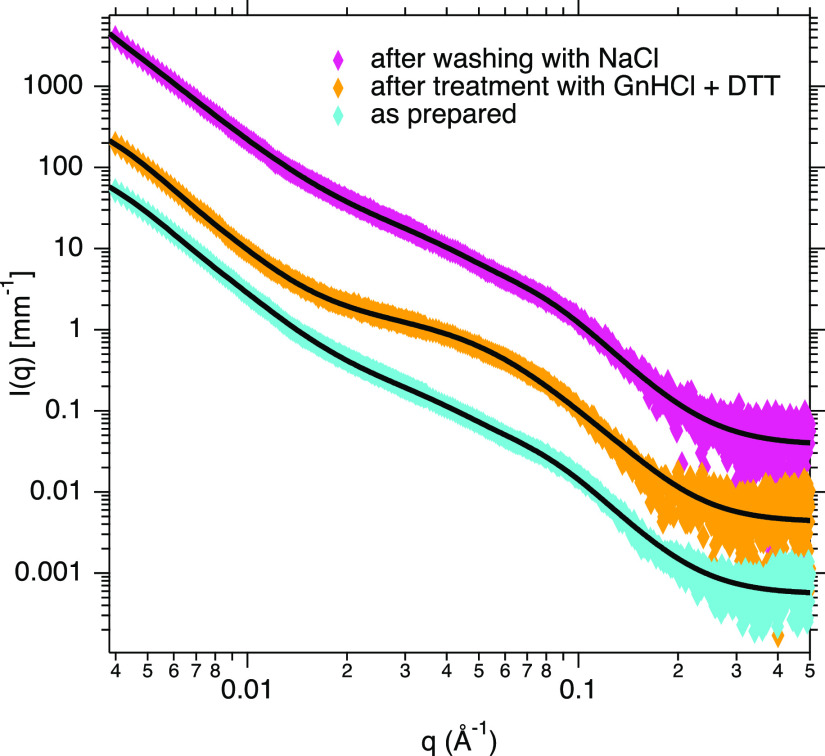
SAXS curves of the as-prepared composite (cyan), after treatment with GnHCl and DTT (orange) and after washing with NaCl (magenta) along with the best fit (black lines) using the Unified fit model. The experimental intensities are shifted along the y-axis for the sake of clarity (offset 1000 mm^–1^). The fitting results are reported in Table S5.

The spectrum of the composite treated with GnHCl and DTT (Figure S5, Table S4) shows an increase in the *Q*^4^/*Q*^3^ ratio from 1.39 to 1.72. This might be a consequence of the fact that lysozyme, when leaving the composite because of its denaturation, causes the detachment of the less condensed and thus more weakly bound parts of the silica. This is also consistent with the structural results obtained through SAXS (Figure 3).

The scattering data were modeled using the Unified model approach.^64^ This approach describes scattering data as composed of multiple dimensional levels with different structural features. Each level is described by a Guinier and an associated Porod power-law regime (see Supporting Information). In particular, the exponential decay in the log–log plot (Guinier region) is directly connected to the average structural size of the scattering level, while the Porod power-law region reflects its fractal dimension. As apparent from the change in shape of the scattering profile after treatment with GnHCl and DTT, the SAXS data show a reduction of the mean radius of the silica primary units from 4 to 3 nm upon protein removal. This result agrees well with the formation of a less compact structure as already suggested by NMR and SEM data. On the contrary, no remarkable changes in the nanostructure of the silica matrix occur after washing with NaCl with a mean radius of the silica primary units of 4 nm as in the starting material.

A direct correlation of ^13^C MAS NMR signals from the protein to ^29^Si MAS NMR signals from the matrix would enable us to reveal an intimate contact between the two components.^65,66^ However, detection of such correlation is necessarily limited since both the ^13^C nuclei in the protein and the ^29^Si nuclei in silica are in natural abundance (1.1% ^13^C and 4.7% ^29^Si) in the complex, reducing the probability of a coupled ^13^C–^29^Si spin pair to 1 in 2000.^65^ Therefore, we have chosen to work on the comparison of the ^1^H traces and to discriminate among the protons that act as polarization sources for the heteronuclear sites. Hence, we have acquired ^1^H-X 2D correlation HETCOR spectra, with wPMLG^67−69^^1^H homonuclear decoupling during the evolution of the indirect ^1^H dimension and cross-polarization (CP)^70,71^ for polarization transfer. To increase the sensitivity, all 2D NMR spectra were processed with multivariate curve resolution (see Supporting Information).^72^

**Figure 4:**
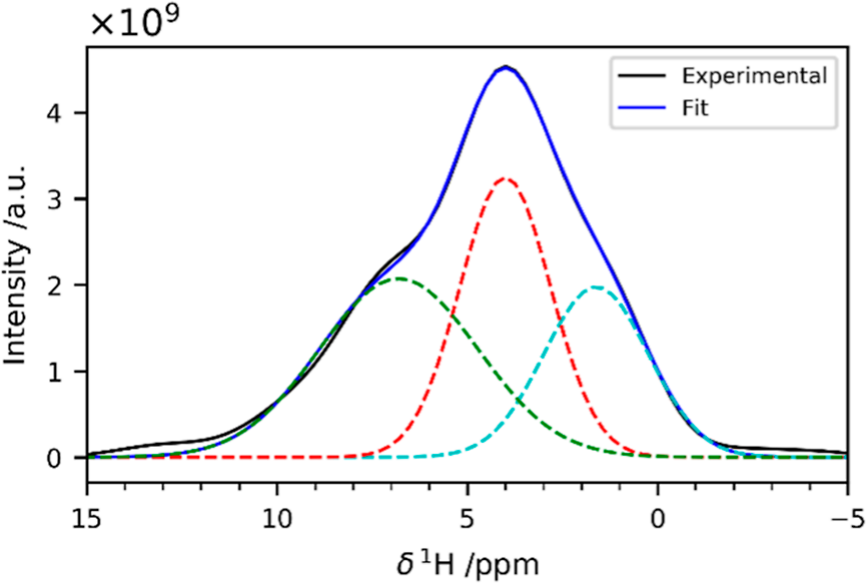
^1^H trace corresponding to the *Q*^4^ sites of the {^1^H}-^29^Si HETCOR acquired with 10 ms of CP contact time on the freeze-dried composite. The signal has been deconvolved with three Gaussian peaks centered at 1.6, 4.0 and 6.8 ppm.

The ^1^H NMR spectra that are obtained projecting the 2D HETCOR on the dry sample along the ^29^Si dimension show cross-polarization coming from at least three unique ^1^H sources. All the traces are rather broad, with features around 1.6, 4, and 7 ppm (a representative example is shown in Figure 4).

**Figure 5:**
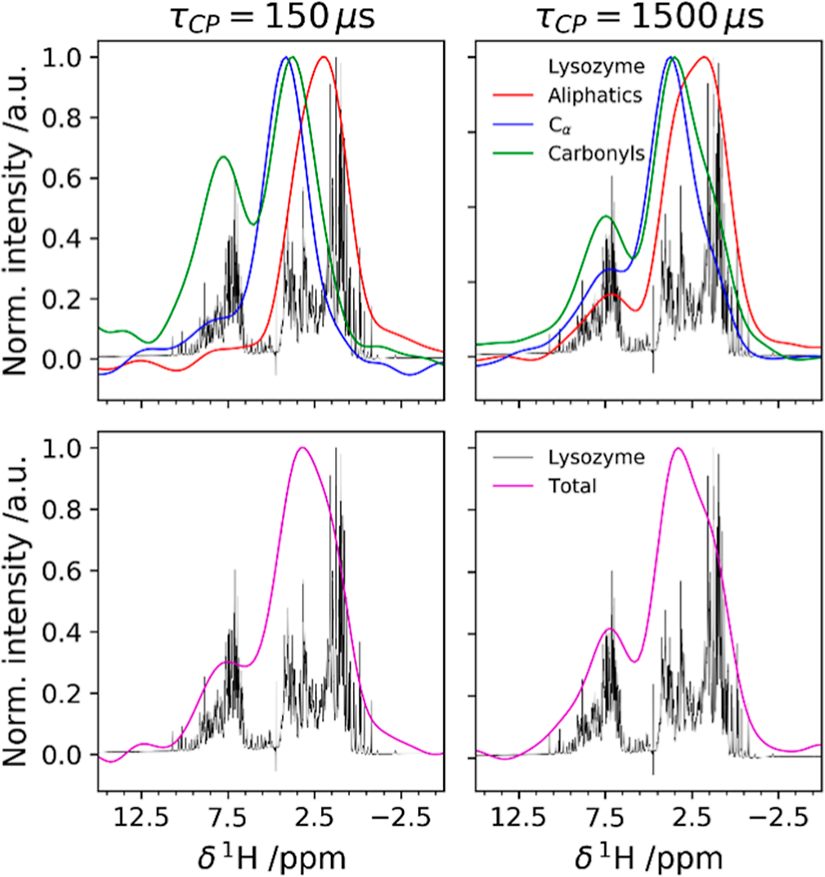
^1^H trace of the {^1^H}–^13^C HETCOR acquired on the freeze-dried composite with 150 μs (left) and 1500 μs (right) of CP contact time. In the top panels, the individual contributions of aliphatic (red), Cαs (blue), and C′s (green) are reported. The integration ranges for the three groups are shown in Figure S9. In the bottom panels, the signal is integrated over all the ^13^C species. The ^1^H spectrum of lysozyme in solution is shown in black in all the spectra.

The first two signals (1.6 and 4.0 ppm) are consistent with signals usually observed for silica: they can be attributed to silanols and physisorbed water, respectively.^73,74^ In biosilica, the feature around 1 ppm has also been ascribed to aliphatic protons from biomolecules.^65^ The feature around 7 ppm deserves more attention. This line is sometimes observed in ^1^H NMR spectra of silica and has been attributed to −OH_2_^+^ species or hydrogen-bonded silanols,^73,75,76^ or water molecules hydrogen-bonded to bridging oxygen atoms.^74,77^ In the specific case of biosilica, it may arise from the protein backbone and/or side chains, although this latter assignment remains elusive.^65^ In the spectrum of the gel obtained in the absence of lysozyme, this feature is completely absent (Figure S7). The fact that this peak, under our experimental conditions, is only observed in the presence of the protein implies that the interaction with the protein alters the behavior of the silica surface. One possibility for further sorting this out is to find out whether the carbon nuclei in the protein and the silicon nuclei in the silica matrix are getting polarized by the same proton source(s) or not. Therefore, the HETCOR experiments were repeated with ^13^C as the target nucleus, still on the dry sample. The ^1^H–^13^C correlation spectra are common for a protein sample where maximal transfer is observed at a shorter mixing time of 150 μs for the directly bonded ^1^H–^13^C spin pairs such as alpha protons (Hα)-alpha carbons (Cα) and at longer times for carbonyl (C′) carbons polarized by amide protons (H_N_) and Hα (Figure 5).

**Figure 6:**
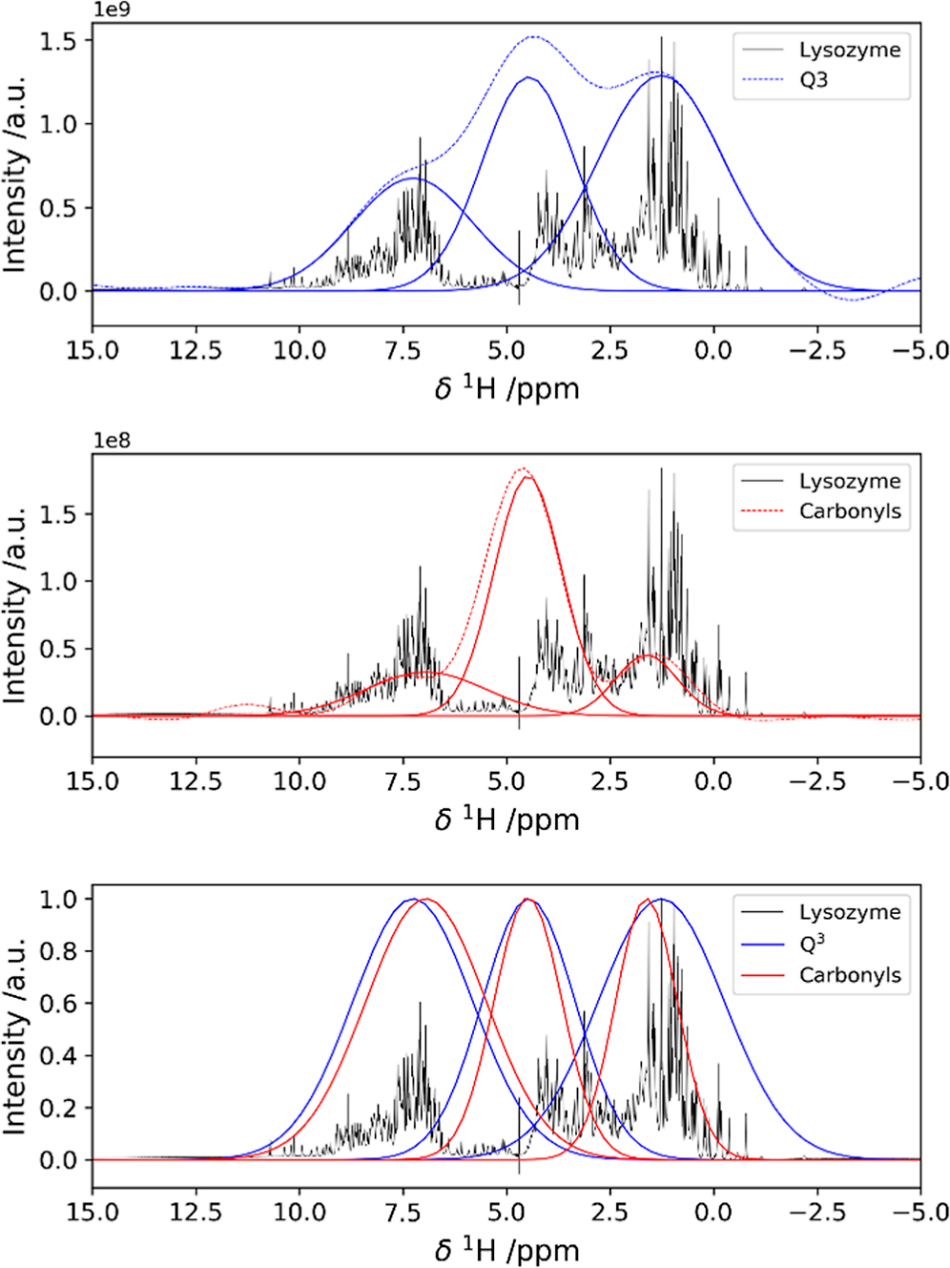
Overlap among the ^1^H polarization sources for ^29^Si and ^13^C HETCOR spectra, revealing the close contact between the protein and the silica matrix. ^1^H trace of the {^1^H}–^29^Si HETCOR acquired with 500 μs of CP contact time on the sample rehydrated with D_2_O. The signal is deconvoluted as a sum of three Gaussian peaks (top). ^1^H trace of the {^1^H}–^13^C HETCOR acquired with 150 μs of CP contact time on the sample rehydrated with D_2_O. The signal is deconvoluted as a sum of three Gaussian peaks (middle). For clarity of representation, the two sets of Gaussian peaks used for the deconvolution of the {^1^H}–^29^Si and the {^1^H}–^13^C HETCOR ^1^H traces are shown together and reported to the same height to highlight the similarity (bottom).

A representative high-resolution ^1^H NMR spectrum of lysozyme in solution is given alongside with the ^1^H trace from the {^1^H}–^13^C HETCOR. We observe that, at low contact times, ^13^C receives magnetization from the closest protein protons and, at longer times, from more distant protons on neighboring bonded atoms (e.g., blue trace shows higher polarization of Cα by H_N_ at 1500 μs). In the {^1^H}–^29^Si HETCOR (integration regions are shown in Figure S7), *Q*^4^^29^Si sites receive magnetization mainly from silanols, whereas *Q*^3^^29^Si sites also receive magnetization from the protons at higher chemical shift values. When longer mixing times are used, the contribution from this proton pool to the *Q*^4^ sites increases (Figure S9). The proton pool at a higher chemical shift is broad and overlaps with the H_N_ proton pool as obtained from the {^1^H}–^13^C HETCOR (see Figure S10, Table S8). If D_2_O is used for rehydration, the ^1^H peak corresponding to amides is reduced (due to D/H exchange) in the traces of the ^13^C NMR spectra but not abolished. Interestingly, the higher-frequency proton pool as detected from the {^1^H}–^29^Si HETCOR sharpens, and the overlap to the H_N_ proton from the {^1^H}–^13^C HETCOR becomes more apparent (Figure 6).

These observations suggest that aromatic, arginine, or lysine side chains or backbone amides are proximate to surface groups in silica. Even under the assumption that the protons at higher chemical shift values only belong to hydrogen-bonded silanols, the intensity of high-chemical shift proton peaks in the {^1^H}–^29^Si HETCOR implies that both H-bonded silanol protons and backbone amide protons are close to *Q*^4^ species on the silica surface.

However, other peaks at lower frequency in the ^1^H dimension, which would be given by *Q*^4^–*K*ε signals, are not observed, unlike other silica–peptide preparations, where clear indication of covalent interaction has been provided.^34^ This is consistent with our experimental observation that the denatured protein is detached from the material, which would not be the case in the presence of a covalent bond.

## Conclusions

In conclusion, lysozyme remains in tight contact with the surface sites of the condensed silica. These interactions do not alter the rigid globular fold of lysozyme appreciably. Chemically induced denaturation is necessary to remove the protein from the composite, pointing again to strong interactions between lysozyme and the inorganic material. Lysozyme is apparently held inside the silica via occluding steric effects rather than by charge–charge interactions (Scheme 1, #2).

## Supporting Information Available

Experimental details for NMR, SEM, and SAXS, SEM micrographs, chemical shifts of ^1^H and ^29^Si species, parameters from the Gaussian deconvolution, ^29^Si direct excitation solid-state NMR spectra, experimental SAXS data, and ^1^H trace of the {^1^H }-^29^Si HETCOR and {^1^H }-^13^C HETCOR (PDF)

## Dedication

This work honors the memory of Shimon Vega, a profound scientist and a dedicated mentor. E.R. and G.G. acknowledge the immense contribution of Shimon Vega to their scientific career and training.

## Abbreviations

TMOS: tetramethylorthosilicate
FESEM: field-emission scanning electron microscope
EDS: energy-dispersive X-ray spectroscopy
wPMLG: windowed phase-modulated Lee-Goldberg
CP: cross-polarization
HETCOR: heteronuclear correlation experiment
CPMG: Carr–Purcell–Meiboom–Gill sequence
DTT: dithiothreitol
GnHCl: guanidinium chloride
MCR: multivariate curve resolution
MAS: magic angle spinning
NMR: nuclear magnetic resonance
SAXS: small-angle X-ray scattering
