# Supplementary material for: Lysozyme is Sterically Trapped Within the Silica Cage in Bioinspired Silica–Lysozyme Composites: A Multi-Technique Understanding of Elusive Protein–Material Interactions
Source: Langmuir. 2022 Jun 23;38(26):8030–7. doi: 10.1021/acs.langmuir.2c00836 (PMC9261187; doi:10.1021/acs.langmuir.2c00836)
Supplement: Supplementary file 1 — la2c00836_si_001.pdf [file la2c00836_si_001.pdf]

Supporting Information

Lysozyme is sterically trapped within the silica cage in Bioinspired Silica-lysozyme Composites: a Multi-technique understanding of elusive Protein-material Interactions

Francesco Bruno[‡,§], Lucia Gigli[‡,§], Giovanni Ferraro[§,°], Andrea Cavallo[Δ], Vladimir K. Michaelis[&], Gil Goobes[%], Emiliano Fratini[§,°] and Enrico Ravera[‡,§,#*]

[‡] Magnetic Resonance Center (CERM), University of Florence, via L. Sacconi 6, 50019 Sesto Fiorentino, Italy;

[§] Department of Chemistry "Ugo Schiff", University of Florence, via della Lastruccia 3, 50019 Sesto Fiorentino, Italy;

° Consorzio per lo Sviluppo dei Sistemi a Grande Interfase (CSGI), via della Lastruccia, 3, 50019 Sesto Fiorentino, Italy;

[Δ] CERTEMA S.c.a.r.l., S.P. Del Cipressino Km 10, 58044 Cinigiano, Italy;

[&] Department of Chemistry, University of Alberta, Edmonton, Alberta T6G 2G2, Canada;

[%] Department of Chemistry, Bar-Ilan University, Ramat Gan 5290002, Israel;

[#] Consorzio Interuniversitario Risonanze Magnetiche di Metalloproteine (CIRMMP), via L. Sacconi 6, 50019 Sesto Fiorentino, Italy.

*Corresponding Author: Enrico Ravera, ravera@cerm.unifi.it

NMR characterization

Solid-state NMR experiments were recorded on a Bruker Avance II spectrometer operating at 700 MHz ^{1}H Larmor frequency (16.4 T), corresponding to 139 MHz ^{29}Si Larmor frequency and 176 MHz ^{13}C Larmor frequency. The spectrometer is equipped with a 3.2 mm BVT MAS probehead in double resonance mode.
The 90° pulse duration on ^{29}Si and ^{13}C for all the analyzed samples were set to 7.5 ms and 3.5 ms respectively. The durations of 90° pulse on ^{1}H were different for the different samples (see Table S1a).
The 1D ^{29}Si spectra in direct excitation were acquired with CPMG echo train acquisition, and then the 24 echoes were separated. Only the first decay has been taken as quantitatively relevant, because the transverse relaxation times are different for the different sites. Interscan delays were set to 3 times the ^{29}Si T_1 of the slowest-relaxing species (Q^4), in order to make the experiments quantitative.
For the 2D {^{1}H}-^{13}C and {^{1}H}-^{29}Si HETCOR experiments, cross-polarization was achieved by matching the k = 1 Hartmann–Hahn condition. The spectral windows for the different nuclei were 60, 248 and 249 ppm for ^{1}H, ^{13}C and ^{29}Si, respectively. During the ^{1}H magnetization evolution under the chemical shift in the indirect dimension of heteronuclear correlation experiments, the PMLG decoupling sequence was used to suppress ^{1}H–^{1}H dipolar couplings.
In these experiments, interscan delay was set to 1.3 T_1 of the ^{1}H relaxation time, shown in Table S1b.
CPMG echo train acquisition has been employed also for {^{1}H}-^{29}Si HETCOR in the direct dimension, and the 24 echoes in each FID were coadded.

Freeze-dried sample	Rehydrated with H_2O	Rehydrated with D_2O	Washed with GnHCl	Washed with NaCl
2.90	2.90	3.17	2.97	3.17

Table S1a. Duration of the ^{1}H 90° pulse in HETCOR experiments, in µs.

Freeze-dried sample	Rehydrated with H_2O	Rehydrated with D_2O	Washed with GnHCl	Washed with NaCl
3.0	2.5	0.8	0.8	3.0

Table S1b. Interscan delay in HETCOR experiments, in seconds.

Small angle X-ray scattering (SAXS) measurements

SAXS measurements were performed on the as-prepared aqueous dispersions of the composite before and after treatment with GnHCl and DTT or washing with NaCl. Scattering profiles were collected using a Xeuss 3.0 HR apparatus (Xenocs, France), equipped with an EIGER2R (1M model) hybrid pixel photon counting detector (Dectris Ltd., Switzerland) consisting of 1,028 x 1,062 pixels with a size of 75 x 75 µm^2, free to move on a rail in a chamber vacuum. The Kα radiation (λ = 1.5406 Å) emitted by a Cu microfocus (30 W) sealed tube operated by the Genix 3D generator. Calibration of the sample to detector distance was performed against silver behenate (d = 58.376 Å).[1] Scattering curves were obtained in the 0.004 and 0.5 Å^{-1} q-range by merging data collected with two different sample to detector distances (i.e. 0.3 and 1.8 m). All the samples were measured under vacuum in 1 mm thick quartz capillary tubes sealed with hot-melting glue. Scattering curves were converted in absolute intensity (mm^{-1}), by measuring the scattering from a 1.16 mm thick glassy carbon reference

specimen in the same experimental conditions.[2,3] Finally the 1-D azimuthal averaged scattering patterns were reduced subtracting the scattering intensity from capillary + water and merging the curves obtained at the two sample-to-detector distances. Data reduction, normalisation and merging were performed by using XSACT (X-ray Scattering Analysis and Calculation Tool) software (Xenocs, France).

Scanning electron microscope (SEM) and energy-dispersive X-ray spectrometry microanalysis (EDS)

All the samples were investigated by a ΣIGMA high-resolution field-emission scanning electron microscope (FESEM, Carl Zeiss) based on the GEMINI column which features a high-brightness Schottky field emission source beam booster and in-lens secondary electron detector. Measurements were performed on uncoated samples mounted on graphite tape with an acceleration potential of 2 kV and at a working distance of about 4 mm.

Energy-dispersive X-ray microanalysis (EDS) analysis was performed using a silicon-drift detector (Oxford Instruments) coupled with SEM at a working distance of 8.5 mm and with an accelerating voltage of 10 kV.

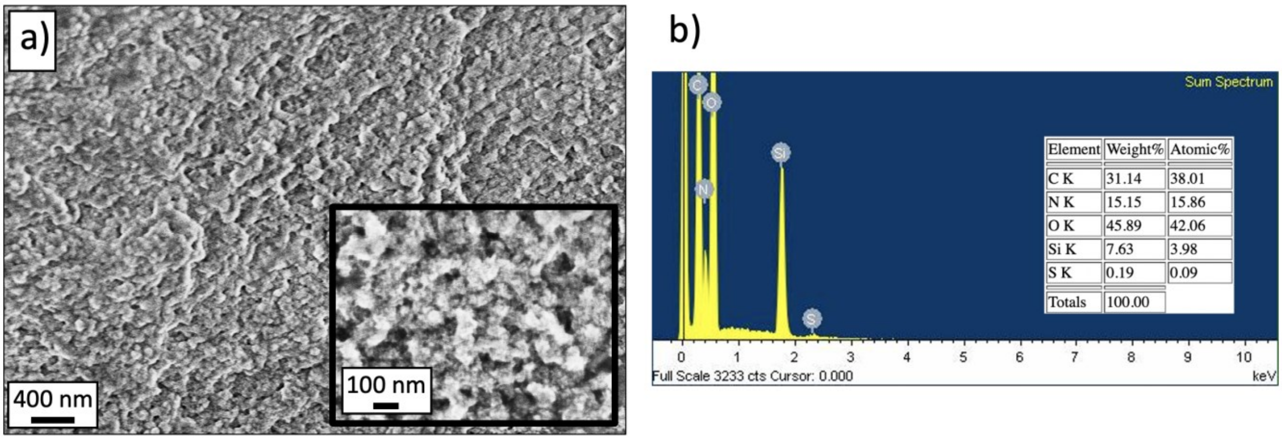

Figure S1. SEM micrographs of the composite after freeze drying at two different magnifications: 50 kX (a) and 200 kX (inset). EDS spectrum of the corresponding sample (b).

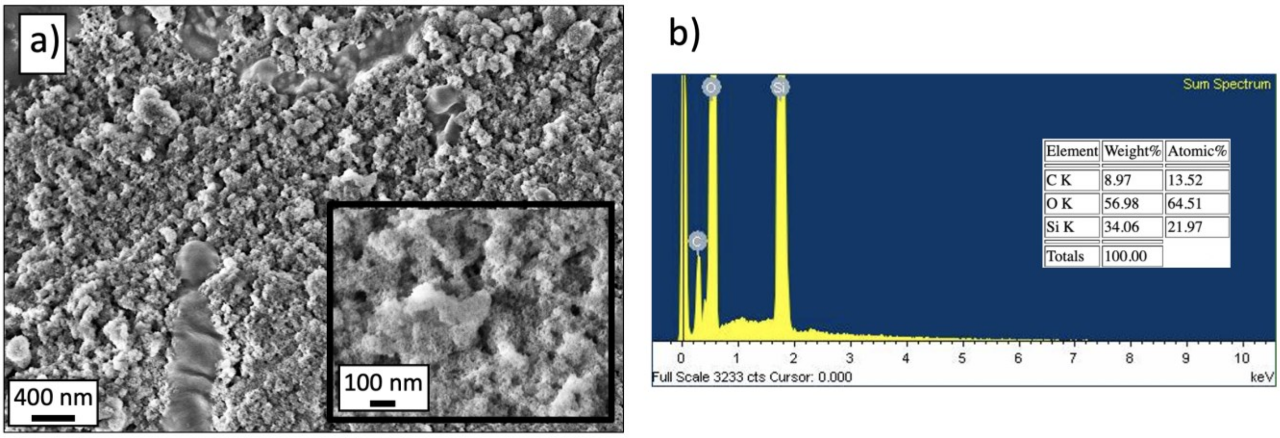

Figure S2. SEM micrographs of the composite after treatment with GnHCl and DTT at two different magnifications: 50 kX (a) and 200 kX (inset). EDS spectrum of the corresponding sample (b).

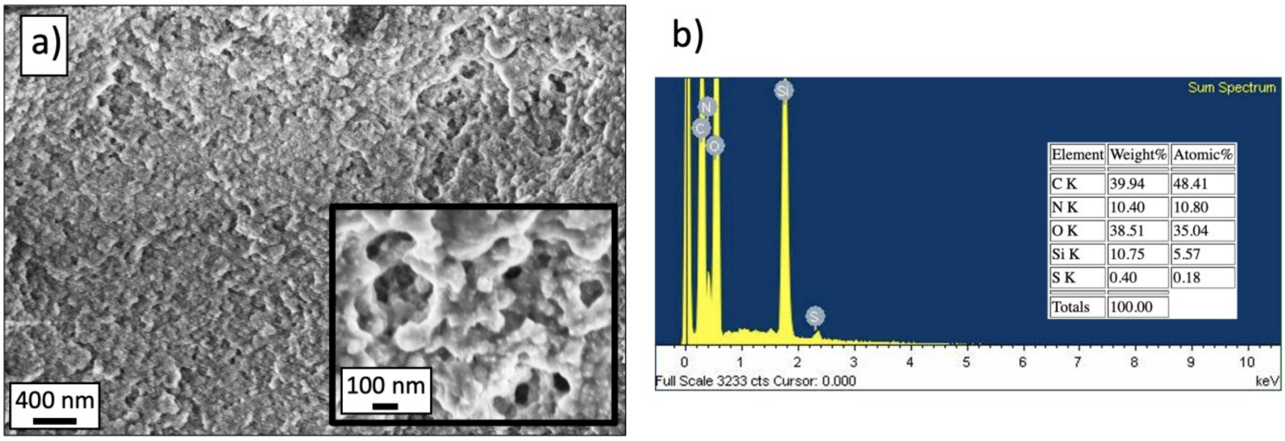

Figure S3. SEM micrographs of the composite after washing with NaCl (a) at two different magnifications: 50 kX and 200 kX (inset). EDS spectrum of the corresponding sample (b).

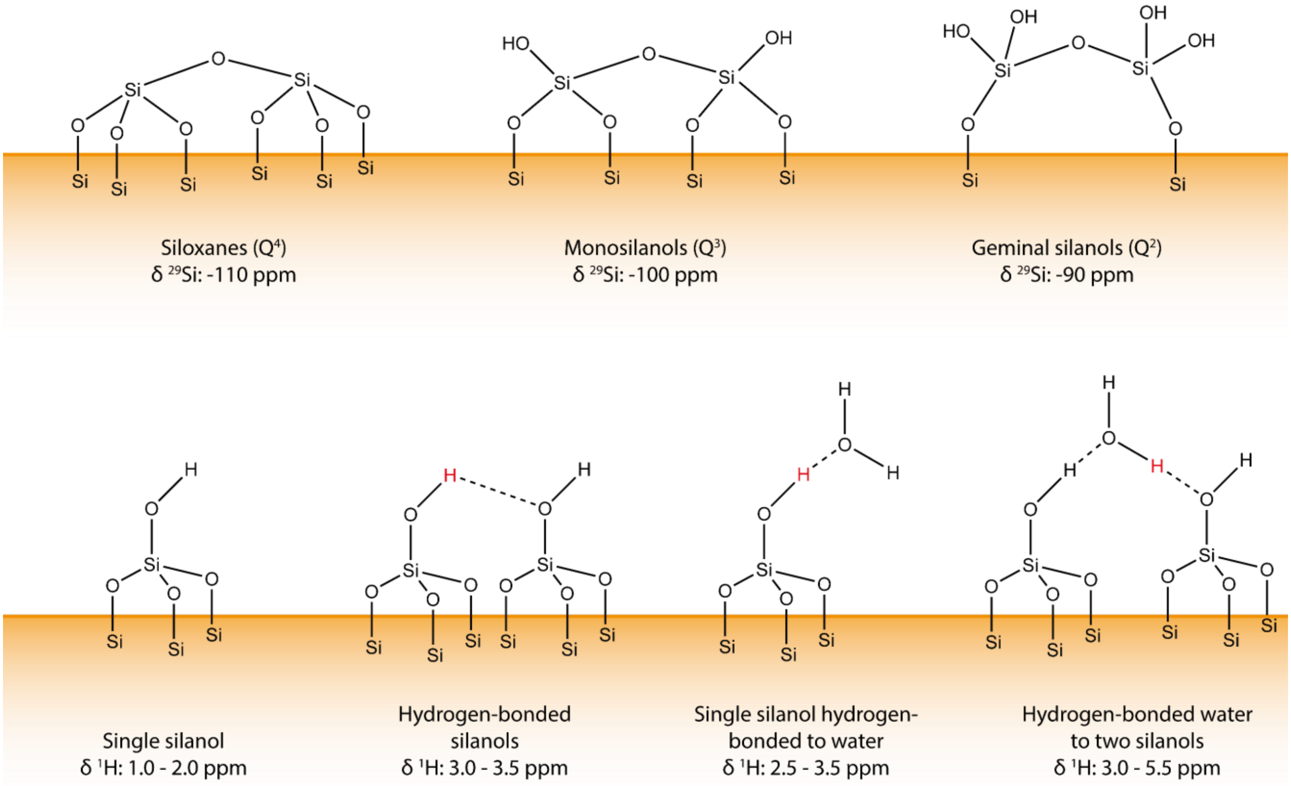

Scheme S1. Chemical shifts of ^{1}H and ^{29}Si species on silica.[4–8]

Site	δ /ppm	σ /Hz	Relative Intensity
Q^2	-93.5	3004	6.2%
Q^3	-103.1	2216	37.5%
Q^4	-112.0	2509	56.3%

Table S2. Parameters from the Gaussian deconvolution of the direct excitation ^{29}Si spectrum of the composite.

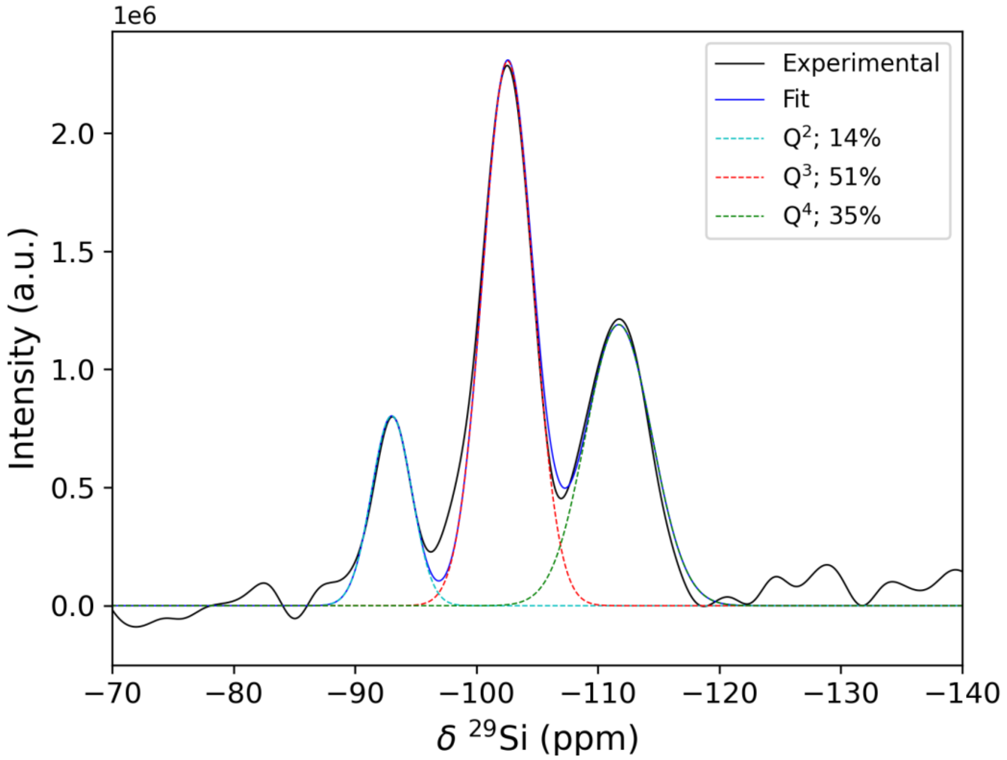

Figure S4. ^{29}Si direct excitation solid-state NMR spectra of silica obtained through condensation of the precursor without the protein. The signal has been deconvoluted using three Gaussian contributions corresponding to Q^2, Q^3 and Q^4 sites.

Site	δ /ppm	σ /Hz	Relative Intensity
Q^2	-93.0	1155	14.1%
Q^3	-102.6	1454	51.1%
Q^4	-111.7	1921	34.8%

Table S3. Parameters from the Gaussian deconvolution of the direct excitation ^{29}Si spectrum of silica obtained through condensation of the precursor without the protein (Figure S4).

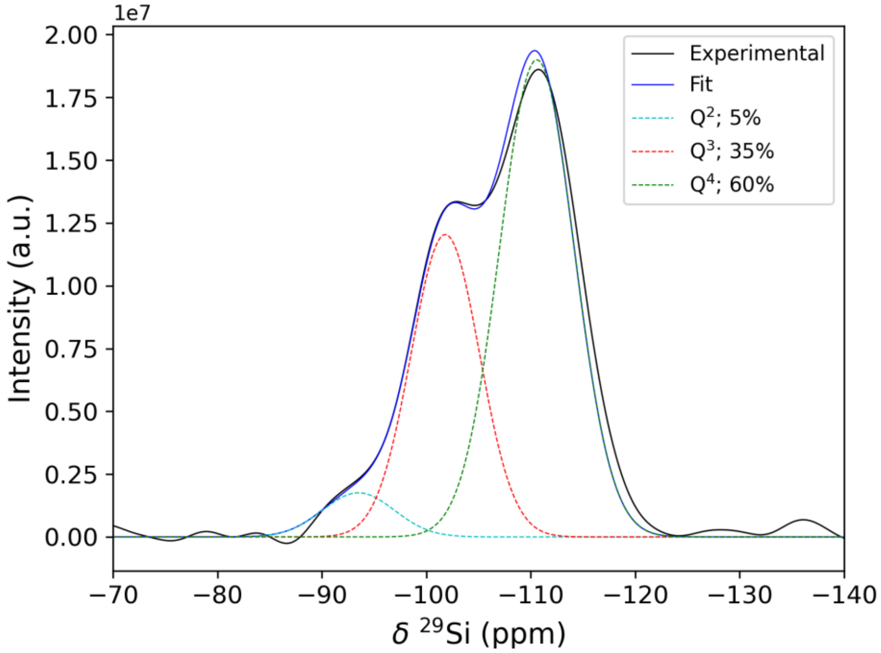

Figure S5. ^{29}Si direct excitation solid-state NMR spectrum of the composite treated with GnHCl and DTT.

**Site**	**δ /ppm**	**σ /Hz**	**Relative Intensity**
**Q2**	-93.5	2409	5.3%
**Q3**	-101.8	2297	34.8%
**Q4**	-110.6	2501	59.8%

Table S4. Parameters from the Gaussian deconvolution of the ^{29}Si direct excitation solid-state NMR spectrum of the composite treated with GnHCl and DTT (Figure S5).

SAXS analysis: Unified fit model

The Unified fit model was proposed by Beaucage in 1995.[9] This approach describes scattering data as composed by multiple dimensional levels with different structural features. Each level is described by a Guinier and an associated Porod power-law regime. It is well known that the first derivation of the Beaucage model in which Guinier and Porod scale factors vary independently can introduce artefacts in the fitting and a detailed discussion on the limitations of this model are reported in the literature.[10] However, this model can be used independently from the shape of the investigated system and for this reason has been widely used for the description of the scattering of randomly distributed structures as spheres, disks, rods and polymer coils.

The final fitting function then is composed by a series of structural levels. The scattering intensity of the level "*i*" is:

$$I_i(q) = G_i e^{-\frac{q^2 R_g^2}{3}} + e^{-\frac{q^2 R_{g(i-1)}^2}{3}} B_i \left\{ \frac{\left[\mathrm{erf}\,(q R_g/\sqrt{6})\right]^3}{q} \right\}^{P_i} \qquad \text{(S1)}$$

where:

- $G = n^2 N_p I_e$

"N_p" is the number of particles in the scattering volume; "I_e" is the scattering factor for a single electron; "n" is the number of electrons in a particle.

- $B = 2\pi N_p S_p \rho_e^2$
  "ρ_e" is equal to n/V_p; "S_p" is the surface area for the particle.

Data analysis was performed using the IRENA modelling package developed by Jan Ilavsky.[11]

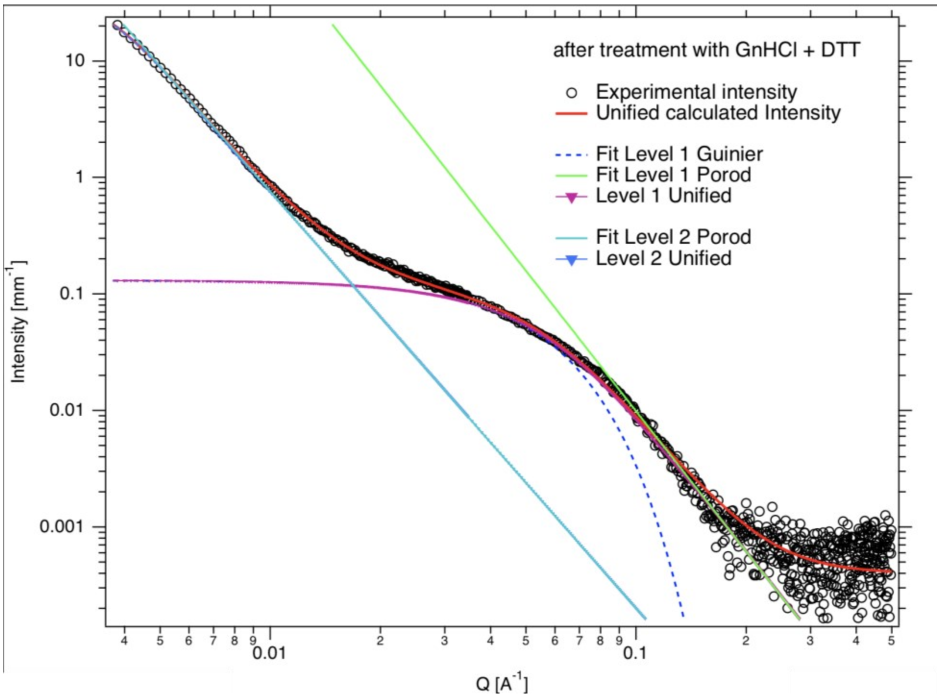

Figure S6. Experimental SAXS data of the sample after treatment with GnHCl + DTT fitted with equation (S1). The black markers are the experimental data, the red curve is the total unified fit, the blue dashed curve represents the Guinier component and green and cyan curves represent the power-law components corresponding to the first and second level, respectively.

	**As-prepared**	**Treatment with GnHCl + DTT**	**Washing with NaCl**
**P (I level, fixed)**	4	4	4
**R_g (nm)**	4.4 (1)	3.4 (1)	4.5 (1)
**P (II level)**	3.3 (1)	3.7 (1)	3.3 (1)
**background**	$4.8 \cdot 10^{-4}$	$3.9 \cdot 10^{-4}$	$3.0 \cdot 10^{-4}$
**χ^2**	2690	1738	1531

Table S5. Parameters extracted from the modelling of SAXS pattern by Unified model. Values in parentheses are standard deviations on the last significant figures.

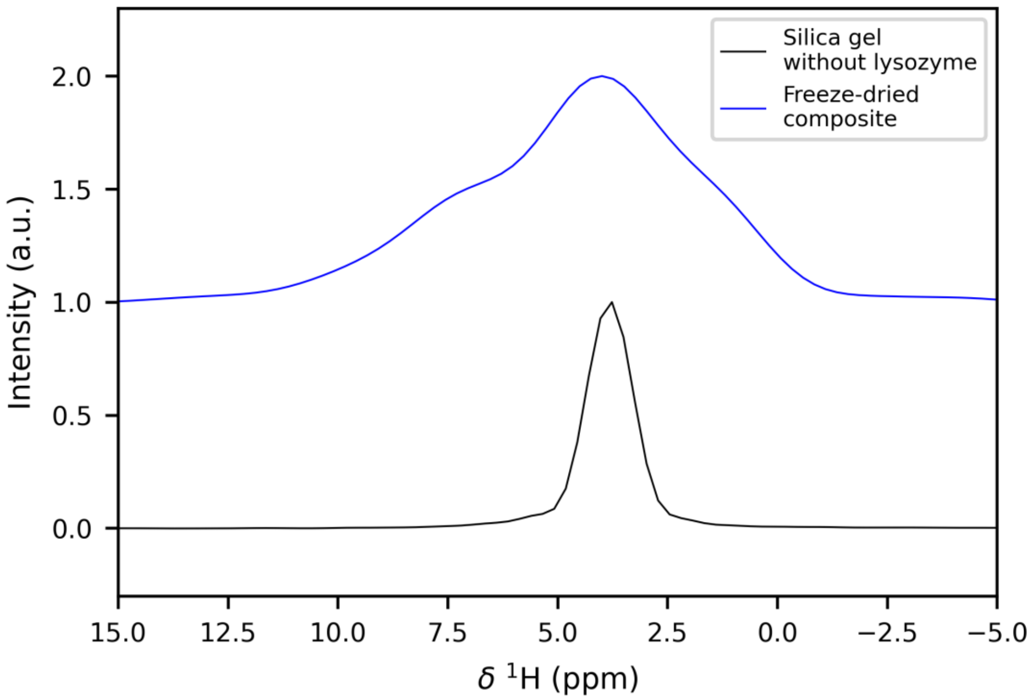

Figure S7. ^{1}H trace of the {^{1}H}-^{29}Si HETCOR acquired with 10 ms of CP contact time on the freeze-dried composite (blue) and on the freeze-dried silica gel obtained in the absence of the protein under otherwise similar conditions (black). The signals are integrated over all Si species.

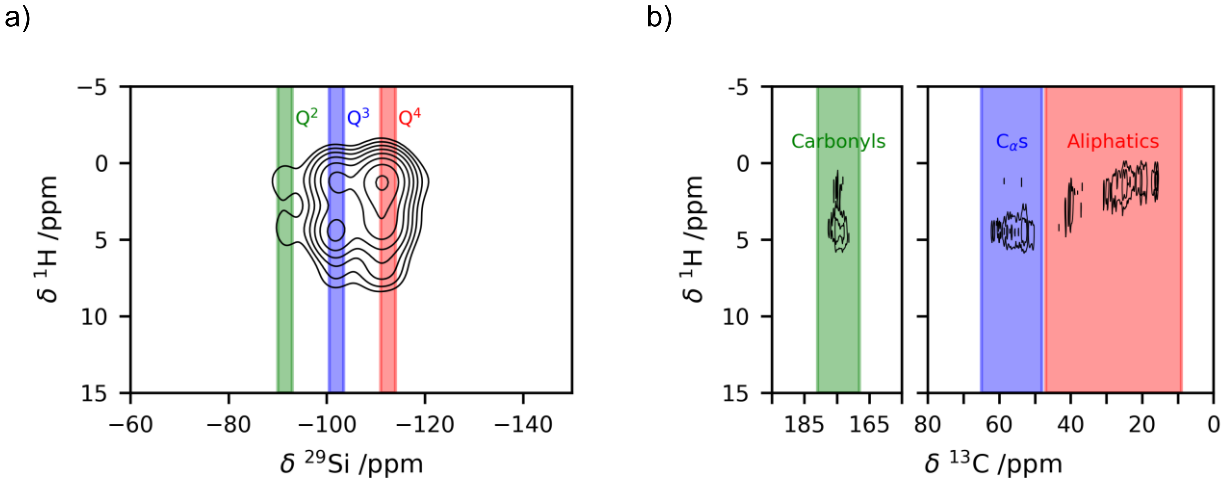

Figure S8. Integration range for ^{1}H traces over the heteronucleus dimension in {^{1}H}-X HETCOR spectra. X is ^{29}Si in the left panel and ^{13}C in the right panel.

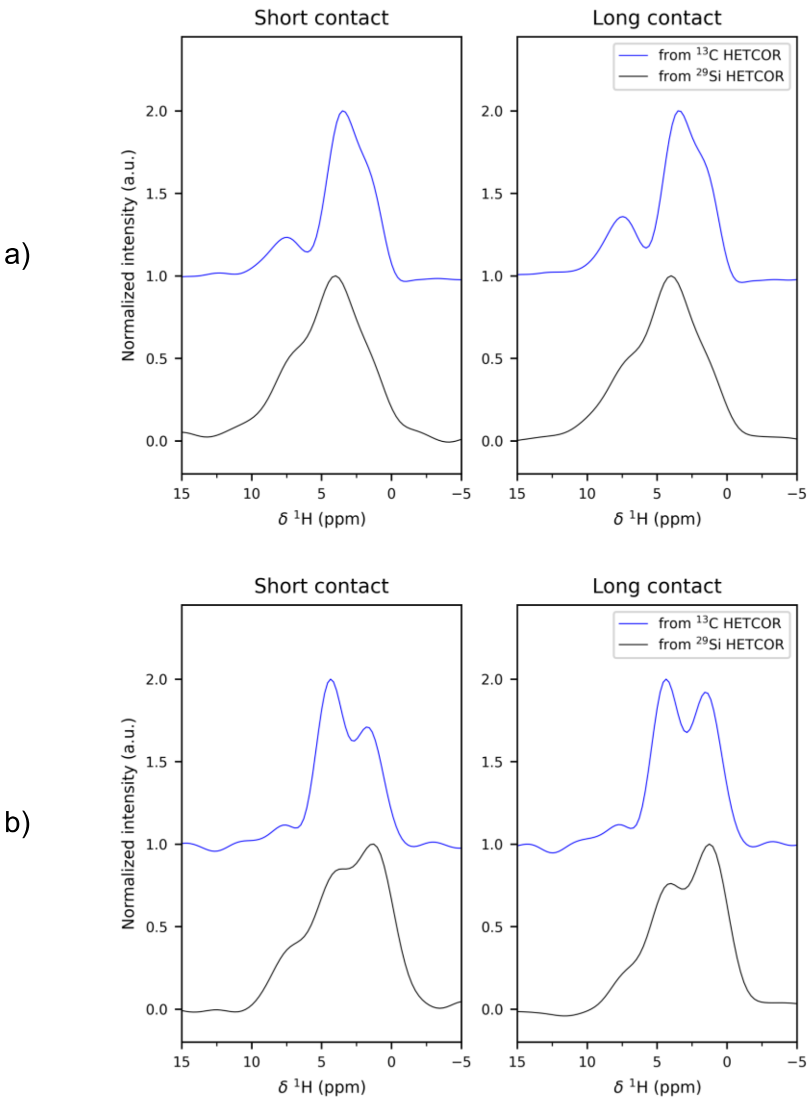

Figure S9. Comparison of the ^1H projection extracted from $\{^1H\}$-^{13}C HETCOR (blue) and $\{^1H\}$-^{29}Si HETCOR (black) spectra, at short (left panel) and long contact (right panel), in the dry sample (top) and in the rehydrated with D_2O sample (bottom).

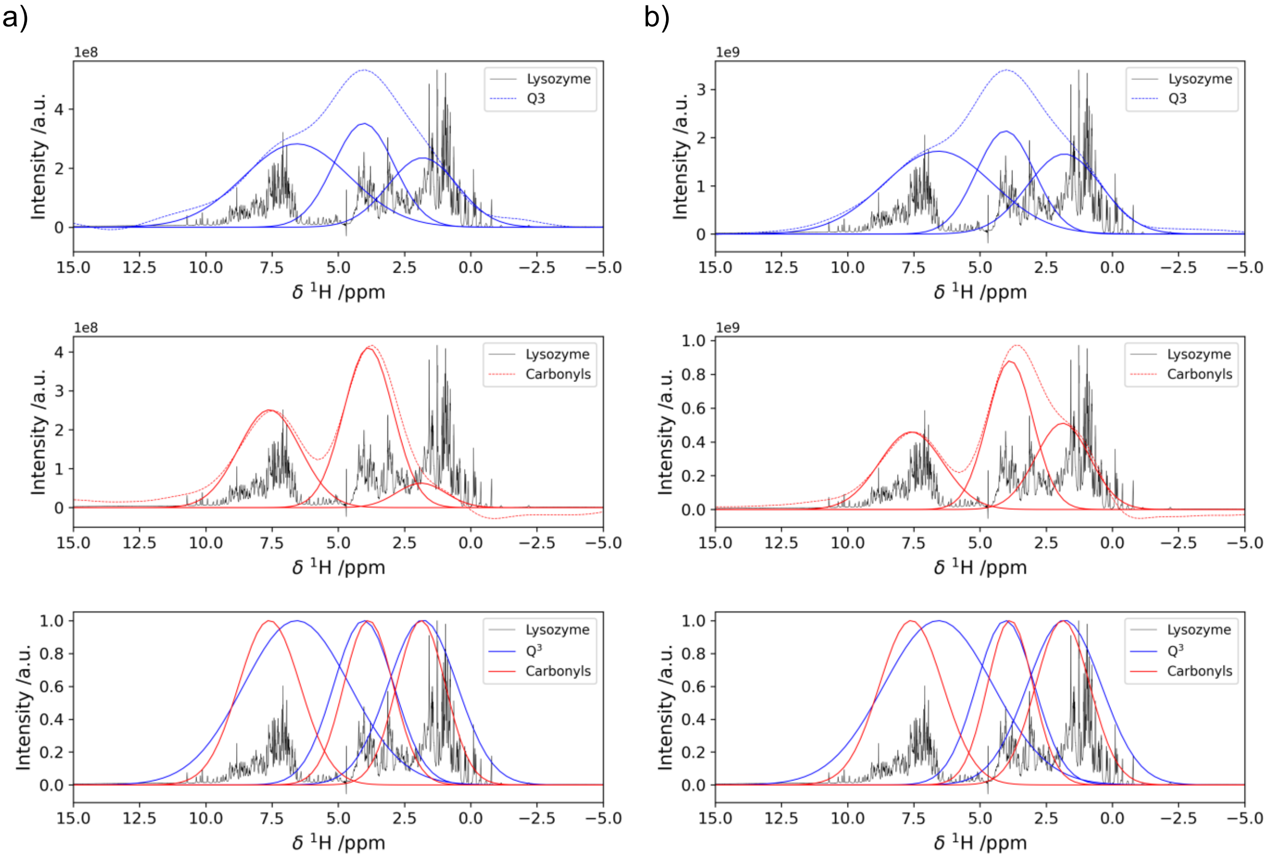

Figure S10. (top) ^{1}H trace of the {^{1}H }-^{29}Si HETCOR acquired with 500 μs (a)/10 ms (b) of CP contact time on the freeze-dried sample. The signal is deconvoluted as a sum of three gaussian peaks. (middle) ^{1}H trace of the {^{1}H }-^{13}C HETCOR acquired with 150 μs (a)/1.5 ms (b) of CP contact time on the freeze-dried sample. The signal is deconvoluted as a sum of three gaussian peaks. (bottom) The two sets of gaussians fitting the {^{1}H }-^{29}Si and the {^{1}H }-^{13}C ^{1}H HETCOR traces, all reported to the same height.

	**Short τ_{CP}**			**Long τ_{CP}**		
**{^{1}H }-^{29}Si site**	**δ /ppm**	**σ /Hz**	**Relative Intensity**	**δ /ppm**	**σ /Hz**	**Relative Intensity**
1	6.6	1403	44%	6.6	1446	43%
2	4.0	797	32%	4.0	767	29%
3	1.8	914	24%	1.8	978	28%
**{^{1}H }-^{13}C site**	**δ /ppm**	**σ /Hz**	**Relative Intensity**	**δ /ppm**	**σ /Hz**	**Relative Intensity**
1	7.6	816	40%	7.6	842	30%
2	3.9	654	52%	3.9	603	41%
3	1.9	684	8%	1.9	718	29%

Table S6. Parameters of the gaussians that fit the spectra in Figure S9.

	Short τ_{CP}		
$\{^1H\}$-^{29}Si site	δ /ppm	σ /Hz	Relative Intensity
1	7.3	1013	22%
2	4.5	787	33%
3	1.3	1068	45%
$\{^1H\}$-^{13}C site	δ /ppm	σ /Hz	Relative Intensity
1	7.0	1019	21%
2	4.5	574	64%
3	1.6	523	15%

Table S7. Parameters of the gaussians that fit the spectra in Figure 6.

(1) Blanton, T. N.; Huang, T. C.; Toraya, H.; Hubbard, C. R.; Robie, S. B.; Louër, D.; Göbel, H. E.; Will, G.; Gilles, R.; Raftery, T. JCPDS—International Centre for Diffraction Data Round Robin Study of Silver Behenate. A Possible Low-Angle X-Ray Diffraction Calibration Standard. *Powder Diffraction* **1995**, *10* (2), 91–95. https://doi.org/10.1017/S0885715600014421.

(2) Zhang, F.; Ilavsky, J.; Long, G. G.; Quintana, J. P. G.; Allen, A. J.; Jemian, P. R. Glassy Carbon as an Absolute Intensity Calibration Standard for Small-Angle Scattering. *Metall Mater Trans A* **2010**, *41* (5), 1151–1158. https://doi.org/10.1007/s11661-009-9950-x.

(3) Allen, A. J.; Zhang, F.; Kline, R. J.; Guthrie, W. F.; Ilavsky, J. NIST Standard Reference Material 3600: Absolute Intensity Calibration Standard for Small-Angle X-Ray Scattering. *J Appl Crystallogr* **2017**, *50* (Pt 2), 462–474. https://doi.org/10.1107/S1600576717001972.

(4) Maciel, G. E.; Sindorf, D. W. Silicon-29 NMR Study of the Surface of Silica Gel by Cross Polarization and Magic-Angle Spinning. *J. Am. Chem. Soc.* **1980**, *102* (25), 7606–7607. https://doi.org/10.1021/ja00545a056.

(5) Liu, C. C.; Maciel, G. E. The Fumed Silica Surface: A Study by NMR. *J. Am. Chem. Soc.* **1996**, *118* (21), 5103–5119. https://doi.org/10.1021/ja954120w.

(6) Grünberg, B.; Emmler, T.; Gedat, E.; Shenderovich, I.; Findenegg, G. H.; Limbach, H.-H.; Buntkowsky, G. Hydrogen Bonding of Water Confined in Mesoporous Silica MCM-41 and SBA-15 Studied by 1H Solid-State NMR. *Chemistry – A European Journal* **2004**, *10* (22), 5689–5696. https://doi.org/10.1002/chem.200400351.

(7) Trébosc, J.; Wiench, J. W.; Huh, S.; Lin, V. S.-Y.; Pruski, M. Solid-State NMR Study of MCM-41-Type Mesoporous Silica Nanoparticles. *J. Am. Chem. Soc.* **2005**, *127* (9), 3057–3068. https://doi.org/10.1021/ja043567e.

(8) Protsak, I. S.; Morozov, Y. M.; Dong, W.; Le, Z.; Zhang, D.; Henderson, I. M. A 29Si, 1H, and 13C Solid-State NMR Study on the Surface Species of Various Depolymerized Organosiloxanes at Silica Surface. *Nanoscale Research Letters* **2019**, *14* (1), 160. https://doi.org/10.1186/s11671-019-2982-2.

(9) Beaucage, G. Approximations Leading to a Unified Exponential/Power-Law Approach to Small-Angle Scattering. *Journal of Applied Crystallography* **1995**, *28* (6), 717–728. https://doi.org/10.1107/S0021889895005292.

(10) Hammouda, B. Analysis of the Beaucage Model. *J Appl Crystallogr* **2010**, *43* (6), 1474–1478. https://doi.org/10.1107/S0021889810033856.

(11) Ilavsky, J.; Jemian, P. R. Irena: Tool Suite for Modeling and Analysis of Small-Angle Scattering. *J Appl Cryst* **2009**, *42* (2), 347–353. https://doi.org/10.1107/S0021889809002222.
